# Detection of cytogenomic abnormalities by OncoScan microarray assay for products of conception from formalin-fixed paraffin-embedded and fresh fetal tissues

**DOI:** 10.1186/s13039-021-00542-5

**Published:** 2021-04-02

**Authors:** Jiadi Wen, Brittany Grommisch, Autumn DiAdamo, Hongyan Chai, Sok Meng Evelyn Ng, Pei Hui, Allen Bale, Winifred Mak, Guilin Wang, Peining Li

**Affiliations:** 1 Department of Genetics, Yale University School of Medicine, New Haven, CT USA; 2 Yale Center for Genome Analysis, Yale University School of Medicine, New Haven, CT USA; 3 Department of Pathology, Yale University School of Medicine, New Haven, CT USA; 4 Department of Women’s Health, Dell Medical School, The University of Texas At Austin, Austin, TX USA

**Keywords:** Products of conception (POC), OncoScan microarray assay (OMA), Formalin-fixed paraffin-embedded (FFPE) tissue, Aneuploidy, Polyploidy, Pathogenic copy number variant (pCNV)

## Abstract

**Background:**

The OncoScan microarray assay (OMA) using highly multiplexed molecular inversion probes for single nucleotide polymorphism (SNP) loci enabled the detection of cytogenomic abnormalities of chromosomal imbalances and pathogenic copy number variants (pCNV). The small size of molecular inversion probes is optimal for SNP genotyping of fragmented DNA from fixed tissues. This retrospective study evaluated the clinical utility of OMA as a uniform platform to detect cytogenomic abnormalities for pregnancy loss from fresh and fixed tissues of products of conception (POC).

**Results:**

Fresh specimens of POC were routinely subjected to cell culture and then analyzed by karyotyping. POC specimens with a normal karyotype (NK) or culture failure (CF) and from formalin-fixed paraffin-embedded (FFPE) tissues were subjected to DNA extraction for OMA. The abnormality detection rate (ADR) by OMA on 94 cases of POC-NK, 38 cases of POC-CF, and 35 cases of POC-FFPE tissues were 2% (2/94), 26% (10/38), and 57% (20/35), respectively. The detected cytogenomic abnormalities of aneuploidies, triploidies and pCNV accounted for 50%, 40% and 10% in POC-CF and 85%, 10% and 5% in POC-FFPE, respectively. False negative result from cultured maternal cells and maternal cell contamination were each detected in one case. OMA on two cases with unbalanced structural chromosome abnormalities further defined genomic imbalances and breakpoints.

**Conclusion:**

OMA on POC-CF and POC-FFPE showed a high diagnostic yield of cytogenomic abnormalities. This approach circumvented the obstacles of CF from fresh specimens and fragmented DNA from fixed tissues and provided a reliable and effective platform for detecting cytogenomic abnormalities and monitoring true fetal result from maternal cell contamination.

## Background

The incidence of pregnancy loss from implantation to clinically recognized spontaneous abortions is approximately 30% [1]. Pregnancy loss of a fetus at any stage is considered a fetal demise, which is further classified as spontaneous abortions or miscarriage when occurring in less than 20 weeks of gestation age and stillbirth for more than 20 weeks. Analysis of genetic defects causing fetal demises has been performed on products of conception (POC) specimens. Karyotyping analysis on culture cells from fresh POC specimens detected numerical aneuploidies and polyploidies as well as structural chromosomal abnormalities in approximately 50% of specimens; for the remaining 50% with a normal karyotype (NK), microarray analysis detected pathogenic copy number variants (pCNVs) and uniparental disomy in approximately 2–4% and 1–2% of specimens, respectively [2–4]. However, POC specimens processed in cytogenetics laboratories encountered culture failure (CF) in 10–20% of cases [2, 3, 5, 6] and maternal cell contamination in 3–22% of cases [4, 6]. A salvage procedure using microarray analysis on POC-CF could detect cytogenomic abnormalities in 35% of cases and thus a strategy of initial karyotyping on culture success cases followed by microarray analysis on POC-NK and POC-CF cases has been implemented [3].

POC specimens preserved in formalin-fixed paraffin-embedded blocks (FFPE) have been recommended by physicians as a resource for genetic testing. However, DNA extracted from FFPE specimens is degraded into small fragments and yields only a small amount. Highly multiplexed molecular inversion probes for targeted single nucleotide polymorphism (SNP) loci could be used for SNP genotyping from fragmented DNA [7, 8]. The Affymetrix OncoScan microarray array (OMA) using molecular inversion probes has been developed and validated for genomic profiling of somatic copy number aberrations of various tumors from FFPE specimens [9, 10]. A pilot study using OMA on 25 archived POC-FFPE specimens showed concordant patterns with previous results by fluorescent in situ hybridization (FISH), which verified its clinical use for constitutional cytogenomic abnormalities [11].

This SNP-based OMA could be used as a uniform platform for DNA extracted from fresh and FFPE fetal tissues, and thus extend the detection of cytogenomic abnormalities from POC-NK and POC-CF to POC-FFPE. In this study, the performance characteristics of OMA were evaluated by the abnormality detection rate (ADR) and the types of cytogenomic abnormalities detected from specimens of POC-NK, POC-CF and POC-FFPE. The results not only justified the clinical utility of OMA for detecting cytogenomic abnormalities and monitoring maternal cell contamination but also provided evidence for an integrated comprehensive approach to better evaluate genetic defects on pregnancy loss from POC.

## Results

During 2018–2020, 94 cases of POC-NK, 38 cases of POC-CF, and 35 cases of POC-FFPE analyzed by OMA were retrieved from the laboratory’s information system [12]. For these 167 cases, the clinical indications included fetal demise (spontaneous abortions and stillbirth) (71.2%, 119/167), suspected fetal anomaly (23.4%, 39/167), preterm premature rupture of membranes (3%, 5/167), and recurrent pregnancy loss (2.4%, 4/167). The maternal age ranged from 20 to 49 years and 46% of them were aged 35 and older. The gestational age ranged from early pregnancy of 6 weeks to stillbirth at third trimester over 37 weeks. The cytogenomic abnormalities detected from OMA on POC-NK, POC-CF and POC-FFPE are summarized in Table 1.

**Table 1:** Cytogenomic abnormalities in POC-NK, CF and FFPE

Abnormalities	POC-NK	POC-CF	POC-FFPE
	n = 94	n = 38	n = 35
Chr Abn. (ADR)	1 (1%)	9 (23%)	19 (54%)
*Aneuploidies*			
Trisomy 4		1	
Trisomy 6			1
Trisomy 8			1
Trisomy 9			3
Trisomy 12			1
Trisomy 13	1		1
Trisomy 14			1
Trisomy 15		1	1
Trisomy 16			3
Trisomy 20			1
Trisomy 21		2	
Trisomy 22		1	
Monosomy X			2
Monosomy 21			2
*Triploidies*			
69,XXX		2	1
69,XXY		2	
70,XXX,+21			1
*pCNV (ADR)*	1 (1%)	1 (3%)	1 (3%)
del(X)(p22.33p22.31)		1	
del(18)(q23)			1
del(22)(q11.21)	1		
Total Abn cases (ADR)	2 (2%)	10 (26%)	20 (57%)

**Fig. 1:**
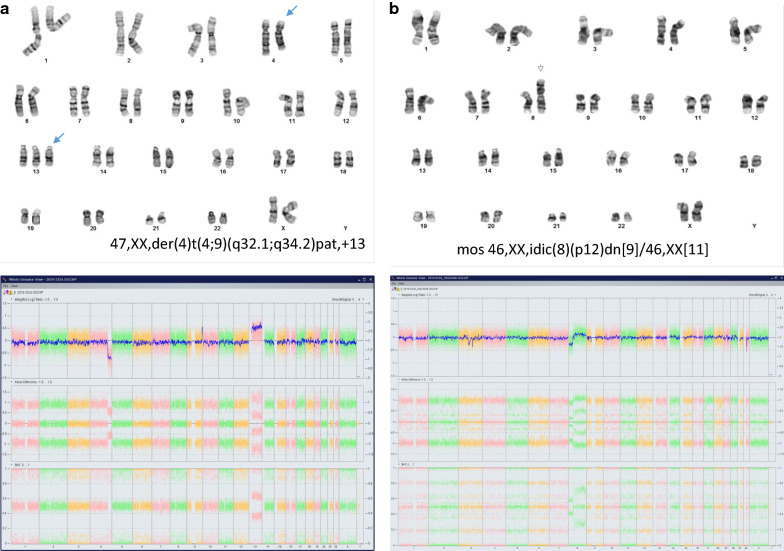
Karyotyping and OMA results from two cases with unbalanced chromosomal rearrangements. **a** Karyotyping result showed a derivative chromosome 4 from an undetermined translocation and trisomy 13 (top panel). Whole genome view of copy number and SNP patterns by OMA detected trisomy 13, a distal deletion of 4q, and a duplication of 9q; which defined the derivative chromosome 4 as the result of a translocation between 4q32 and 9q34.3 (bottom panel). **b** Karyotype shows an isodicentric chromosome 8 and OMA shows a distal deletion of 8p and a duplication of remaining 8p and 8q

Of the 94 POC-NK cases, 43 cases were male and 51 cases were female. Two cases (2%, 2/94) were detected with an abnormal result. One case was detected with a normal female karyotype from cultured villi cells. Re-evaluation by OMA using DNA from FFPE showed a male sex pattern and three copies of chromosome 13. These findings indicated a false negative result from karyotyping of cultured maternal cells. Another case with a normal female karyotype was detected with a 2.586 Mb deletion at 22q11.21 (chr22:18876425_21462601x1) by OMA and confirmed by FISH. This deletion includes the *TBX1* gene and is diagnostic for DiGeorge/velocardiofacial syndrome. Region of homozygosity (ROH) in about 3% of genome was detected in two cases. OMA was also performed on four cases with unbalanced or balanced chromosomal rearrangements (Table 2). In one case with a trisomy 13 and a derivative chromosome 4 from a 4q32.1/9q34.2 translocation of paternal origin, OMA result confirmed trisomy 13, a 33.540 Mb deletion of 4q32.1q35.2, a 4.916 Mb duplication of 9q34.2q34.3, and also absence of genes at breakpoints 4q32.1 and 9q34.2. In another case with a mosaic pattern of an isodicentric chromosome 8, OMA confirmed a 33.090 Mb deletion of 8p23.3p12, a 113.1 Mb duplication of 8p12q24.3, and likely an interruption of the *FUT10* gene at the fusion point. Further parental chromosome analyses showed normal results; thus, this idic(8) in the fetus was de novo. Figure 1 shows the karyotype and OMA results for these two cases. Two cases with a balanced translocation had a normal result by OMA, indicating an absence of cryptic imbalance in this translocation.

**Table 2:** OMA results on four cases with balanced/unbalanced chromosomal rearrangements

Case	MA	Kayotypes	OMA results (GRCh37/hg19)
1	37	47,XX,der(4)t(4;9)(q32.1;q34.2)pat,+13	arr 4q32.1q35.2(157375250_190915650)x1, 9q34.2q34.3(136859048_141054761)x3,(13)x3
2	27	mos 46,XX,idic(8)(p12)dn[9]/46,XX[11]	arr 8p23.3p12(172416_33262869)x1, 8p12q24.3(33262870_146363022)x3
3	36	46,XY,t(1;10)(p32;q11.2)	Normal
4	41	46,XY,t(1;12)(q32;q24.1)	Normal

*MA* maternal age

Of the 38 cases of POC-CF, 16 cases were male and 22 were female, and 10 cases (26%, 10/38) were detected with an abnormal result. The abnormal findings included a triploidy in four cases, trisomy 21 in two cases, trisomy 4 and 15 each in one case, trisomy 22 with a deletion of 21p11.2q11.2 in one case, and a 7.614 Mb deletion of Xp22.33p22.31 (chrX:177941_7792383x0) in a male case. The case of trisomy 22 with a deletion of 21p and proximal region of 21q likely resulted from a 21q/22p translocation. The couple experienced unexplained recurrent pregnancy loss, and chromosome analysis found normal results. Of the ten abnormal cases in POC-CF, aneuploidies, triploidies and pCNV accounted for 50% (5/10), 40% (4/10) and 10% (1/10), respectively.

Of the 35 cases of POC-FFPE, 14 cases were male and 21 were female, and 20 cases (57%, 20/35) were detected with an abnormal result. The abnormal findings included a triploidy in two cases, monosomy X in two cases, trisomy 16 in three cases, trisomy 9 in three cases, monosomy 21 in two cases, trisomies 6, 8, 12, 13, 14, 15 and 20 each in one case, and a 2.975 Mb deletion at 18q23 (chr18:75032971_78007784x1) in one case. maternal cell contamination was noted in one case with a normal female pattern and multiple B-allele frequency in OMA. Of the 20 abnormal cases in POC-FFPE, aneuploidies, triploidies and pCNV accounted for 85% (17/20), 15% (2/20) and 5% (1/20), respectively.

## Discussion

Initial karyotyping on culture success cases followed by aCGH analysis on specimens of POC-NK and POC-CF has been implemented in Yale Clinical Cytogenetics Lab; compiled results from a five-year case series and relevant studies through literature review demonstrated an overall ADR of 4.6% for pCNVs in POC-NK and of 35% for chromosomal abnormalities and 3.7% for pCNVs in POC-CF [3]. The results from this study by OMA showed an ADR of 1% for chromosome abnormality and 1% for pCNV in POC-NK, of 23% for chromosome abnormalities and 3% for pCNVs in POC-CF, and of 54% for chromosomal abnormalities and 3% for pCNVs in POC-FFPE (Table 1). The trisomy 13 detected by OMA in a case with a normal female karyotype most likely from maternal cells was considered a false negative result in the POC-NK. The ADR from POC-CF were comparable with previous observation [3]. The ADR from POC-FFPE were also consistent with 50% chromosomal abnormalities and 2–4% pCNV observed in large case series of POC [2–4]. The higher detection rate for chromosome abnormalities in POC-FFPE compared with POC-CF may be due to the difference of gestational age of these two groups of samples. The POC-FFPE cases were exclusively from first trimester miscarriages whereas POC-CF are mixed samples of all gestational ages. Likely due to the limited number of cases in this study, abnormalities of uniparental disomy were not detected but consanguinity of half first cousins by about 3% of multiple ROH in the genome was noted in two cases and maternal cell contamination was noted in one case.

The overall results indicated that chromosomal abnormalities accounted for approximately 50% and 23–35% of genetic defects in POC-FFPE and POC-CF, respectively. The causal effect of aneuploidy and polyploidy for fetal demises have been well recognized. Microarray analysis has been effective to detect all numerical chromosomal abnormalities and to define genomic imbalances and rearrangement break points from unbalanced structural chromosomal abnormalities. Identifying carriers of a balanced translocation and tracking unbalanced derivative chromosomes in their fetuses has been a standard procedure for recurrent pregnancy loss [4, 13, 14]. Earlier studies suggested that POC-CF may be related to particular chromosomal imbalances such as autosomal monosomies which could be incompatible with cell proliferation under in vitro cell culture [15, 16]. The studies of POC-CF by previous aCGH analysis [3] and current OMA did not find any particular chromosomal or genomic imbalances. In contrast, the pCNV accounted for only 1–3% of genetic defects in POC-NK, POC-CF and POC-FFPE. OMA detected three pCNV of a deletion at 22q11.2, a deletion at 18q23, and a deletion at Xp22.33p22.31. A study of 22,451 miscarriage samples reported a higher incidence of 22q11.2 deletion in the miscarriages than the reported prevalence in the general population, indicating that the 22q11.2 deletion could be a causal factor for miscarriage [17]. Deletions of the distal region of 18q are associated with chromosome 18q deletion syndrome with wide phenotypic variability [18]. Deletion and duplication at Xp22.33p22.31 involving the *SHOX* and *STS* genes have been detected in POC [3, 4]. Terminal pCNV could also cause genomic instability and resulted in complex chromosomal rearrangement during mitosis [19]. Despite effort in identifying critical genes and loci for human early development from pCNVs, interpretation of causal effect of pCNV for spontaneous abortions and stillbirth could still be a challenge [3, 20, 21]. Further identification of recurrent pCNV on large case series or cohorts of specific fetal anomalies and functional characterization of causal mechanisms for pregnancy loss from these pCNV are required.

**Fig. 2:**
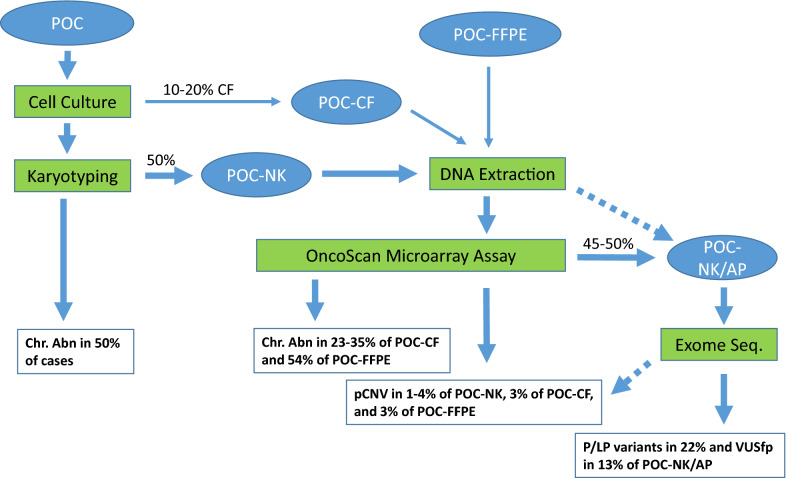
Flow chart for laboratory processing of POC specimens and diagnostic yield from karyotyping, microarray and exome sequencing analyses. The abnormality detection rate for chromosomal abnormality, pCNV, monogenic pathogenic/likely pathogenic (P/LP) variants and variant of uncertain significance favor pathogenic (VUSfp) was from this study and references #3 and #23. Solid arrow for current workflow and dash arrow for an alternative option (*Chr. Abn* chromosomal abnormality, *CF* culture failure, *FFPE* formalin-fixed paraffin-embedded, *NK* normal karyotype, *POC* products of conception, *AP* absence of pCNV)

Genetic testing and result interpretation follow technology-driven and evidence-based approaches [22]. A recent study using exome sequencing on a cohort of POC-NK with absence of pCNV showed ADR of 22% for pathogenic and likely pathogenic variants and 13% for variants of uncertain significance favor pathogenic; which extended the genetic etiology for POC to monogenic Mendelian genes and validated its clinical utility [23]. Therefore, a more comprehensive approach should be initial karyotyping on culture success cases followed by microarray analysis on POC-NK, POC-CF and POC-FFPE and exome sequencing on remaining cases absent cytogenomic abnormalities. Since pCNV accounted for only 2–4% of genetic defects in POC and exome sequencing can detect pCNV, a direct exome sequencing on POC-NK could be a more cost-effective option. This comprehensive approach could potentially detect genetic etiology of chromosomal abnormalities, pCNV and monogenic variants for up to 80% of POC cases (Fig. 2). More accurate and efficient diagnosis of various genetic defects in POC could provide better genetic counseling and clinical management for patients experiencing pregnancy losses.

## Conclusions

This study demonstrated that OMA could be used as a uniform platform for detecting cytogenomic abnormalities from fresh and fixed fetal tissues of POC. OMA can detect cytogenomic abnormalities of aneuploidy, triploidy, unbalanced chromosomal rearrangements and pCNV and ROH for uniparental isodisomy and consanguinity and monitor maternal cell contamination. A comprehensive approach with initial karyotyping followed by OMA and subsequent exome sequencing could significantly improve the diagnostic yield for patients with pregnancy loss.

## Methods

Specimens of POC, including fresh chorionic villi, skin or other fetal tissues and FFPE tissue blocks, were submitted to Yale Clinical Cytogenetics Laboratory for cytogenomic analysis. Initial karyotyping analysis on cultured success cases from fresh fetal tissues followed by oligonucleotide array comparative genomic hybridization (aCGH) analysis on cases of POC-NK and POC-CF was adopted as previously described [3]. DNA extraction from POC-FFPE specimen was performed by hydrothermal pressure coupled with chaotropic salt column purification [24]. A validation study using both aCGH (Agilent 8 × 60 K, 60,000 probes) and OMA (Affymetrix, 220,000 SNP probes) on 15 POC specimens showed concordant results. Since 2018, OMA has been used for genomic DNA samples extracted from specimens of POC-NK, POC-CF, and POC-FFPE. Cases with a balanced or an unbalanced chromosomal rearrangement by karyotyping were also analyzed by OMA to further define breakpoints and genomic imbalances.

OMA was performed at the Yale Center for Genome Analysis using Affymetrix OncoScan FFPE assay kit and following manufacturer’s protocol with integrated quality control measures. Briefly, 80 ng of genomic DNA from POC specimens was used as a template to anneal a panel of molecular inversion probes. The annealing mix was split for AT and GC reactions. The gap between the probes and template was filled in by DNA polymerase and linked by ligase to form circularized probes. Un-ligated probes and single-stranded DNA were destroyed by exonuclease and washed out. Circular probes were released, cleaved and amplified using universal primers by first polymerase chain reaction (PCR). These PCR mixtures were divided for second PCR for the incorporation of either biotinylated AT or GC. These two PCR mixtures were digested by HaeIII, then denatured and hybridized to microarray chip. The results were analyzed using the Chromosome Analysis Suite version 3.3 (Affymetrix, Santa Clara, CA), in which the log2 ratio for copy number changes and B-allele frequency for allelic patterns were visualized. The quality metrics for OMA are mapd ≤ 0.3 and ndSnpQc ≥ 26. The reporting criteria included pCNVs (deletions and duplications) larger than one megabase (Mb), ROH larger than 10 Mb or involving chromosomes known to have uniparental isodisomy associated phenotypes, and multiple ROH encompassing 3% or more of the autosomal DNA complement. However, smaller changes with pathogenic potential will also be reported, while larger changes that are well-documented benign variants will not be reported.

The laboratory procedures and result analysis followed the standards and guidelines of American College of Medical Genetics and Genomics (ACMG) and the regulations of Clinical Laboratory Improvement Act (CLIA) [25–27]. The genomic coordinates for detected chromosomal imbalances and pCNVs were based on the February 2009 Assembly (GRCh37/hg19) of the UCSC Human Genome browser (http://genome.ucsc.edu/). The performance characteristics of OMA on POC-NK, POC-CF and POC-FFPE were evaluated by the abnormality detection rate (ADR) and the types of cytogenomic abnormalities. This study was determined as a chart review retrospective analysis and deemed exempted from Institutional Review Board approval and granted waiver of consent based on the policy of the Yale University Institutional Review Board.

## Data Availability

The data of this study are available from the corresponding authors upon reasonable request.

## Abbreviations

OMA: OncoScan microarray assay
SNP: Single nucleotide polymorphism
pCNV: Pathogenic copy number variants
POC: Products of conception
NK: Normal karyotype
CF: Culture failure
FFPE: Formalin-fixed paraffin-embedded
ADR: Abnormal detection rate
ROH: Region of homozygousity
PCR: Polymerase chain reaction
aCGH: Array comparative genomic hybridization

## References

[1] Wilcox AJ, Weinberg CR, O'Connor JF, Baird DD, Schlatterer JP, Canfield RE, Armstrong EG, Nisula BC (1988). Incidence of early loss of pregnancy. N Engl J Med.

[2] Levy B, Sigurjonsson S, Pettersen B, Maisenbacher MK, Hall MP, Demko Z, Lathi RB, Tao R, Aggarwal V, Rabinowitz M (2014). Genomic imbalance in products of conception: single-nucleotide polymorphism chromosomal microarray analysis. Obstet Gynecol.

[3] Zhou Q, Wu SY, Amato K, DiAdamo A, Li P (2016). Spectrum of cytogenomic abnormalities revealed by array comparative genomic hybridization on products of conception culture failure and normal karyotype samples. J Genet Genomics.

[4] Wang Y, Cheng Q, Meng L, Luo C, Hu H, Zhang J, Cheng J, Xu T, Jiang T, Liang D, Hu P, Xu Z (2017). Clinical application of SNP array analysis in first-trimester pregnancy loss: a prospective study. Clin Genet.

[5] van den Berg MM, van Maarle MC, van Wely M, Goddijn M (2012). Genetics of early miscarriage. Biochim Biophys Acta.

[6] Zhang T, Sun Y, Chen Z, Li T (2018). Traditional and molecular chromosomal abnormality analysis of products of conception in spontaneous and recurrent miscarriage. BJOG.

[7] Hardenbol P, Banér J, Jain M, Nilsson M, Namsaraev EA, Karlin-Neumann GA, Fakhrai-Rad H, Ronaghi M, Willis TD, Landegren U, Davis RW (2003). Multiplexed genotyping with sequence-tagged molecular inversion probes. Nat Biotechnol.

[8] Hardenbol P, Yu F, Belmont J, Mackenzie J, Bruckner C, Brundage T, Boudreau A, Chow S, Eberle J, Erbilgin A, Falkowski M, Fitzgerald R, Ghose S, Iartchouk O, Jain M, Karlin-Neumann G, Lu X, Miao X, Moore B, Moorhead M, Namsaraev E, Pasternak S, Prakash E, Tran K, Wang Z, Jones HB, Davis RW, Willis TD, Gibbs RA (2005). Highly multiplexed molecular inversion probe genotyping: over 10,000 targeted SNPs genotyped in a single tube assay. Genome Res.

[9] Foster JM, Oumie A, Togneri FS, Vasques FR, Hau D, Taylor M, Tinkler-Hundal E, Southward K, Medlow P, McGreeghan-Crosby K, Halfpenny I, McMullan DJ, Quirke P, Keating KE, Griffiths M, Spink KG, Brew F (2015). Cross-laboratory validation of the OncoScan FFPE Assay, a multiplex tool for whole genome tumour profiling. BMC Med Genomics.

[10] Singh RR, Mehrotra M, Chen H, Almohammedsalim AA, Sahin A, Bosamra A, Patel KP, Routbort MJ, Lu X, Ronald A, Mishra BM, Virani S, Medeiros LJ, Luthra R (2016). Comprehensive screening of gene copy number aberrations in formalin-fixed, paraffin-embedded solid tumors using molecular inversion probe-based single-nucleotide polymorphism array. J Mol Diagn.

[11] Gliem TJ, Aypar U (2017). Development of a chromosomal microarray test for the detection of abnormalities in formalin-fixed, paraffin-embedded products of conception specimens. J Mol Diagn.

[12] Xiang B, Li P, Hemingway SS, Qumsiyeh M (2006). CytoAccess, a relational laboratory information management system for a clinical cytogenetics laboratory. J Assoc Genet Technol.

[13] Li P, Pomianowski P, DiMaio MS, Florio JR, Rossi MR, Xiang B, Xu F, Yang H, Geng Q, Xie J, Mahoney MJ (2011). Genomic characterization of prenatally detected chromosomal structural abnormalities using oligonucleotide array comparative genomic hybridization. Am J Med Genet A.

[14] Wei Y, Gao X, Yan L, Xu F, Li P, Zhao Y (2012). Prenatal diagnosis and postnatal follow up of partial trisomy 13q and partial monosomy 10p: a case report and review of the literature. Case Rep Genet.

[15] Fritz B, Hallermann C, Olert J, Fuchs B, Bruns M, Aslan M, Schmidt S, Coerdt W, Müntefering H, Rehder H (2001). Cytogenetic analyses of culture failures by comparative genomic hybridisation (CGH)-Re-evaluation of chromosome aberration rates in early spontaneous abortions. Eur J Hum Genet.

[16] Lebedev IN, Ostroverkhova NV, Nikitina TV, Sukhanova NN, Nazarenko SA (2004). Features of chromosomal abnormalities in spontaneous abortion cell culture failures detected by interphase FISH analysis. Eur J Hum Genet.

[17] Maisenbacher MK, Merrion K, Pettersen B, Young M, Paik K, Iyengar S, Kareht S, Sigurjonsson S, Demko ZP, Martin KA (2017). Incidence of the 22q11.2 deletion in a large cohort of miscarriage samples. Mol Cytogenet..

[18] Strathdee G, Sutherland R, Jonsson JJ, Sataloff R, Kohonen-Corish M, Grady D, Overhauser J (1997). Molecular characterization of patients with 18q23 deletions. Am J Hum Genet.

[19] Chai H, Grommisch B, DiAdamo A, Wen J, Hui P, Li P (2019). Inverted duplication, triplication and quintuplication through sequential breakage-fusion-bridge events induced by a terminal deletion at 5p in a case of spontaneous abortion. Mol Genet Genomic Med.

[20] Reddy UM, Page GP, Saade GR, Silver RM, Thorsten VR, Parker CB, Pinar H, Willinger M, Stoll BJ, Heim-Hall J, Varner MW, Goldenberg RL, Bukowski R, Wapner RJ, Drews-Botsch CD, O'Brien BM, Dudley DJ, Levy B, NICHD Stillbirth Collaborative Research Network (2012). Karyotype versus microarray testing for genetic abnormalities after stillbirth. N Engl J Med..

[21] Chen Y, Bartanus J, Liang D, Zhu H, Breman AM, Smith JL, Wang H, Ren Z, Patel A, Stankiewicz P, Cram DS, Cheung SW, Wu L, Yu F (2017). Characterization of chromosomal abnormalities in pregnancy losses reveals critical genes and loci for human early development. Hum Mutat.

[22] Wei Y, Xu F, Li P (2013). Technology-driven and evidence-based genomic analysis for integrated pediatric and prenatal genetic evaluation. J Genet Genomics.

[23] Zhao C, Chai H, Zhou Q, Wen J, Reddy UM, Kastury R, Jiang Y, Mak W, Bale AE, Zhang H, Li P (2020). Exome sequencing analysis on products of conception: a cohort study to evaluate clinical utility and genetic etiology for pregnancy loss. Genet Med.

[24] Zhong H, Liu Y, Talmor M, Wu B, Hui P (2013). Deparaffinization and lysis by hydrothermal pressure (pressure cooking) coupled with chaotropic salt column purification: a rapid and efficient method of DNA extraction from formalin-fixed paraffin-embedded tissue. Diagn Mol Pathol.

[25] South ST, Lee C, Lamb AN, Higgins AW, Kearney HM, Working Group for the American College of Medical Genetics and Genomics Laboratory Quality Assurance Committee (2013). ACMG Standards and Guidelines for constitutional cytogenomic microarray analysis, including postnatal and prenatal applications: revision 2013. Genet Med..

[26] Rehder CW, David KL, Hirsch B, Toriello HV, Wilson CM, Kearney HM (2013). American College of Medical Genetics and Genomics: standards and guidelines for documenting suspected consanguinity as an incidental finding of genomic testing. Genet Med.

[27] Riggs ER, Andersen EF, Cherry AM, Kantarci S, Kearney H, Patel A, Raca G, Ritter DI, South ST, Thorland EC, Pineda-Alvarez D, Aradhya S, Martin CL (2020). Technical standards for the interpretation and reporting of constitutional copy-number variants: a joint consensus recommendation of the American College of Medical Genetics and Genomics (ACMG) and the Clinical Genome Resource (ClinGen). Genet Med.

